# Disruptions in loading and aeration impact effluent chlorine demand during biological greywater recycling

**DOI:** 10.1016/j.wroa.2020.100087

**Published:** 2021-01-21

**Authors:** Christopher Ziemba, Pragnya Sharma, Theresa Ahrens, Eva Reynaert, Eberhard Morgenroth

**Affiliations:** a Eawag: Swiss Federal Institute of Aquatic Science and Technology, 8600, Dübendorf, Switzerland; b ETH Zürich, Institute of Environmental Engineering, 8093, Zürich, Switzerland

**Keywords:** Permeate quality, Hand washing water, Decentralized, Chlorination, Biologically activated membrane bioreactor (BAMBi), Gravity-driven membrane (GDM) treatment

## Abstract

Greywater recycling systems designed for high-quality applications, such as hand washing, must deliver microbially safe and aesthetically acceptable water under the challenging operating conditions present where such systems are needed most urgently. As chlorination is the most popular strategy for reducing bacterial concentrations in greywater, understanding chlorination in the context of disruptive and challenging operation is essential to designing robust treatment. In this study, we have examined how disruptions through overall increased loading, interrupted aeration and increased ammonia loading have impacted the chlorine demand of the water produced by a greywater recycling system. We also presented concentrations of significant chemicals that contributed to this chlorine demand. The results indicate that a 1 d period with 8 times (8x) the normal design loading produced a peak chlorine demand of 0.74 mg Cl_2_/L, which is approximately double the baseline value. While this chlorine demand can be overcome by adding more chlorine, tests involving disruptions in aeration or feeding additional ammonia into the bioreactor produced much greater increases (>30x). The risks of increased chlorine demand on microbial safety can be overcome by limiting ammonia inputs to the system, providing backup systems to ensure sufficient aeration, or through additional anti-bacterial measures that do not depend on maintaining residual chlorine.

## Highlights

• Increasing loading up to 8x for 1 d produces manageable Cl_2_ demand.
• Disrupted aeration produces high Cl_2_ demand from NH_3_ and DOC.
• Increasing NH_3_ loading produces significant Cl_2_ demand due to NH_3_ passage.

## Graphical abstract

**Figure:**
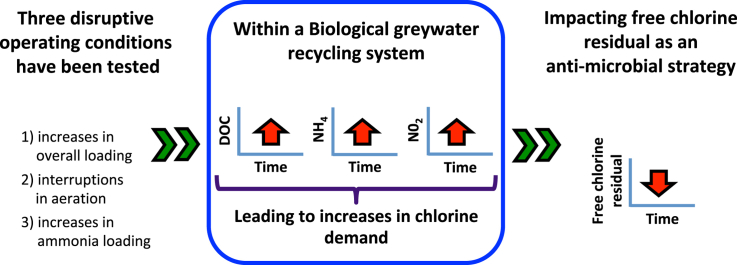

## 1 Introduction

Decentralized biological greywater treatment systems able to reliably recycle greywater for high-quality applications, such as hand washing, promise a valuable opportunity to expand global access to safe hygiene. Such systems could be most impactful in parts of the world where access to any water is limited and the costs of centralized water treatment and distribution is economically prohibitive. Unfortunately, existing greywater research has largely focused on delivering water for less-demanding applications such as toilet flushing (Shi et al., 2018; Zhu et al., 2018) or irrigation (Busgang et al., 2018). Recent research has specifically investigated post-treatment technologies that are able to improve effluent water quality for higher-quality applications, such as hand washing water or showering (Dubowski et al., 2020; Gassie and Englehardt, 2017; Nguyen et al., 2017; Ziemba et al, 2019, 2020). These works have demonstrated the feasibility of decentralized and self-contained systems, appropriately sized for one home, community installation or medical facility, that will be filled initially with water and then repeatedly treat this water, provide it for washing or showering, recollect it and repeat this cycle. What remains to be explored is how challenging or disruptive operation might affect water quality and, in turn, microbial safety. The small scale of decentralized systems can make them sensitive to variability in loading (the dirt, soap, skin and other materials added to the greywater) in terms of amount and composition. The application of decentralized systems in remote environments or areas with unreliable electricity access may impose additional challenges, such as the inability to quickly replace broken components or frequent power interruptions. Understanding the implications of challenging and disruptive operating conditions on system performance is crucial to implement safe and effective biological greywater recycling for high-quality applications.

Critical analysis of current greywater technologies in practice has concluded that biologically-based treatments can be the most environmentally friendly (in terms of chemical requirements and energy demand), while also providing high-quality effluent with small footprint requirements (Boyjoo et al., 2013). These advantages have caused biological treatment processes to become broadly-accepted in the greywater treatment field (Pidou et al., 2007), but the importance of understanding how different greywater treatment systems perform under variable conditions has also been noted (Ghunmi et al., 2011). Biological systems may exhibit reduced ability, relative to physio-chemical systems, to adapt quickly to changes in operation or environment, and may require specific conditions such as nutrient-balance or minimum oxygen concentrations.

Any type of operational disruptions (increased loading, altered composition of loading and interrupted aeration) can result in reduced biological activity and accumulation of undesirable materials within the system. This accumulation can create an unpleasant (color, odor or feel of the water), ineffective (hands cannot get clean) or unsafe (ineffective bacteria control) experience for the user. While the factors that contribute to a negative user perception are evident, how disruptions affect microbial safety may be more complex. The most common form of greywater disinfection is chlorination (Boyjoo et al., 2013). Chlorination is a particularly effective disinfection strategy because maintaining a free chlorine residual within a water system combats bacterial growth during storage. Studies conducted with a variety of greywater treatment technologies have concluded from results that a free chlorine residual of 1 mg Cl_2_/L achieves acceptable anti-bacteria control (Abdel-Kader, 2013; Winward et al., 2008; Ziemba et al., 2019). Maintaining a chlorine residual in the treated water requires overcoming the losses of chlorine to volatilization, externally-driven reactions (e.g. photoreduction) and the chlorine demand of the water to which the chlorine is added. Chlorine demand consists of all chemical constituents within the water that would rapidly react with chlorine. Ammonia, nitrite and general organic carbon compounds can all exhibit significant chlorine demand. Throughout this study, the term ammonia refers to the combination of ammonia and ammonium species. While the chlorine demand of ammonia and nitrite can be estimated stoichiometrically, we are not aware of any study that has measured the chlorine demand of organic materials in treated greywater. Our limited understanding of how disruptive operation contributes to chemical accumulation, increases chlorine demand and opposes the efficacy of microbial control through chlorination, represents a significant knowledge gap that prevents us from developing robust biological greywater recycling systems.

One important task for the further development of greywater recycling systems for high-quality applications like handwashing will be the definition of quality targets. Over the past years, there has been an increasing number of water reuse frameworks targeting lower-quality reuse applications such as irrigation or toilet flushing. However, as yet, only the ISO 30500 standard for non-sewered sanitation systems explicitly includes handwashing as an application for recycled water, though it does not include recommendations or requirements specifically related to chlorinating systems. Microbial safety for high-quality applications will require log removal performance for greywater treatment similar to drinking water treatment (ISO 30500, WHO 2017). A better understanding of the factors affecting the chlorine demand in greywater recycling systems designed for high-quality applications can inform the developers of new or revised water reuse frameworks in the establishment of quality targets for greywater reuse systems that include a final chlorination step.

The impacts of disruptive operation were examined in this study using a biologically activated membrane bioreactor (BAMBi), which has been developed specifically for decentralized greywater recycling (Künzle et al., 2015; Ravndal et al., 2015; Ziemba et al., 2020). Inside the BAMBi, treatment is conducted by bacteria, either in the bulk liquid phase or in a biofilm that develops on an immersed ultrafiltration membrane module, and within a granular activated carbon (GAC) filter that follows the membrane. Gentle aeration directly below the membrane module promotes aerobic conversion in external areas of the biofilm. BAMBi reactors operating at steady state were subject to a series of 1 d disruptive events that could be encountered in practice, (i) an increase in loading, (ii) an increase in ammonia loading, (iii) a loss of aeration. The chlorine demand of the produced water was measured or stoichiometrically estimated from relevant measured compounds before, during and after each disruption. Increasing aeration during increased loading has also been tested as a potential mitigation strategy. By understanding the mechanisms and the magnitude of each potential disruption we can make informed design decisions for biological systems that utilize chlorination and help advance decentralized hand washing water recycling into the real (and disruptive) world.

## 2 Methods and materials

### 2.1 Greywater treatment system

**Fig. 1:**
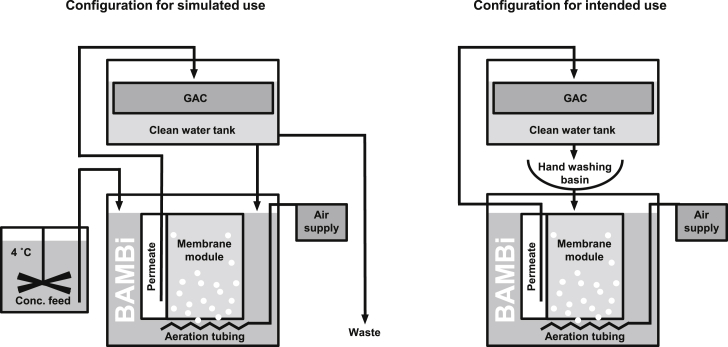
Process schematic for the biologically activated membrane bioreactor (BAMBi) as tested during simulated usage and as intended for actual usage. In all testing, BAMBi was paired with a granular activated carbon (GAC) filter. Clean water from the clean water tank (after GAC) is mixed with concentrated synthetic hand washing water (Table 1) and recycled back to the BAMBi.

The flow of water through the greywater recycling system is illustrated in Fig. 1. A concentrated nutrient-balanced synthetic hand washing greywater was prepared and stored at 4 °C with 10 rpm mixing. Under normal operation, a total of 3 L/d of the concentrated feed was pumped into the BAMBi in a series of 40 feedings, evenly distributed throughout the day. The daily loading of the 3 L of concentrated synthetic feed represents the loading (soap, skin, material on hands) that would be introduced to a total of 60 L of water during hand washing (1.5 L per hand washing event). The BAMBi itself consisted of a standing sandwich membrane module (Microclear MCXL, Newterra, Ontario, Canada) featuring a 150 kDa polyethersulfone ultrafiltration membrane (Microdyn-Nadir, Wiesbaden, Germany) placed into a 52 L wastewater tank (Fig. 1). The operating volume of the BAMBi was approximately 42 L. The membrane was positioned in the tank such that the top of the membrane matched the water level. Aeration was provided directly below the membrane module with a pair of aeration tubes that created bubbles approximately 3–4 mm in diameter (Air Curtain 90 cm, Guangdong Risheng, Shenzhen, China). Under normal operation, the rate of aeration was 6 L/min. This aeration creates regions within the BAMBi that permit aerobic processes (such as nitrification) and anaerobic processes (such as denitrification) within the same simple reactor.

No additional pressure was supplied to drive water through the membrane beyond the pressure head of water on the feed side of the membrane. Water that passed through the biofilm and through the membrane was collected in a permeate reservoir and then pumped to the clean water storage tank at 5 min intervals. Water entering the storage tank first passed vertically down through a 6 L granular activated carbon (GAC) filter (Norit 830, ∼1.5 mm grain diameter, Cabot, Boston, MA, USA) submerged in the storage tank. The GAC was a mixture of new GAC (25%) combined with a balance of GAC that had been previously used in a similar greywater treatment system for ∼6 months. After passing through the GAC, the water entered the larger storage tank and was then returned to the BAMBi by an overflow. The overflow maintained a constant volume of 17.5 L of water in the storage tank (including water in the GAC). The passage of water from the clean water storage tank back to the BAMBi represented water that would be available for hand washing in an operational system. Extra water was pumped out of the clean water storage tank (and discharged) to compensate for the concentrated greywater feed and maintain a constant system volume. Though the standard loading corresponded to 60 L of daily water usage, water was recirculated through the system at approximately the rate of membrane flux (∼120 L/d).

The feeding, permeate and water removal pumps were activated using automation software (Codesys, 3S-Smart Software Solutions GmbH, Kempten, Germany and CitectSCADA, Schneider Electric, Rueil-Malmaison, France). Aeration was controlled manually by a rotameter (Analyt-MTC, Colmar, France). The BAMBi was initially started with tap water, inoculated with 0.5 L of activated sludge from a municipal wastewater treatment plant, and operated for approximately 30 d before stable performance was achieved.

### 2.2 Synthetic hand washing water feed

The recipe for the concentrated synthetic hand washing water feed is presented in Table 1. This recipe is based on the expected inputs to hand washing greywater and the nutrient-balancing strategy presented previously (Ziemba et al., 2018). In this study, the nutrient demands were based on the estimate that 1/3 of the carbon in assimilated substrate will be converted to new biomass, and that the production of this biomass will require nutrient-balancing. This yield of 1/3 biomass carbon/substrate carbon was reduced from the yield of 1, which was used previously and we have also removed kaolin as an ingredient (Ziemba et al., 2018). The concentrated feed water was prepared in tap water. The tap water in Zürich has been measured as containing 0.7 mg/L N, 5 mg/L S, 50 mg/L Ca, 1.2 mg/L K, 7 mg/L Mg, <0.005 mg/L P, Fe, Zn and <0.0005 mg/L Mn and Cu (City of Zürich, 2016). Mo and Co were assumed to be zero.

**Table 1:** 20x concentrated synthetic hand washing water recipe as prepared in tap water.

Compound	Formula	Concentration (mg/L)
Sodium dodecyl sulfate (SDS)	NaC_12_H_25_SO_4_	4200
Glycerol	C_3_H_8_O_3_	1500
Humics (approximate formula)	C_9_H_8_Na_2_O_4_	51
Lactic acid	C_3_H_6_O_3_	22
Sodium nitrate	NaNO_3_	379
Ammonium nitrate	NH_4_NO_3_	357
Disodium hydrogen phosphate	HNa2O_4_P⋅2H_2_O	269
Potassium chloride	KCl	29.8
Iron chloride tetrahydrate	Cl_2_Fe⋅4H_2_O	27.8
Manganese chloride tetrahydrate	Cl_2_Mn⋅4H_2_O	0.12
Cobalt chloride hexahydrate	Cl_2_Co⋅6H_2_O	0.017

The specific values provided in Table 1 reflect what must be added to the tap water to get the desired nutrient content. The humic acid was a naturally collected material (53680, Sigma-Aldrich, St. Louis, Missouri, USA). All other chemicals were reagent grade or better. Diluting the 20x concentrated synthetic hand washing water to 1x strength (as used in the system), contributed an organic carbon concentration of ∼130 mg TOC/L and an ammonia concentration of 3.1 mg N/L.

### 2.3 Disruption experiments

Three types of disruptions were examined (Table 2), consisting of increasing loading (scenarios 1–4), turning off the aeration (scenario 5), or changing the composition of the feed (scenario 6). Increasing the loading was accomplished by increasing the amount of feed concentrate pumped into the BAMBi and removing more treated water to maintain constant volume. The aeration flow rate was set to zero as a disruption. The composition was changed by replacing the standard synthetic greywater feed with a feed that was modified with a spike of 1000 mg/L NH_4_–N in the form of NH_4_Cl. This spike in the feed delivered an additional 3 g of NH_4_–N over the course of the disruption, which corresponds to the amount of ammonia that would be released from the hydrolysis of 400 mL of fresh human urine (Udert et al., 2006). The total ammonium dosing during the disruption was increased to approximately 17x the standard feed. This corresponds after dilution to an ammonia concentration of 53.1 mg N/L in the diluted feed compared to the standard 3.1 mg N/L.

**Table 2:** Disruption testing.

Scenario #	Type of disruption	24 h disruption	Mitigation strategy	Results
1	increased loading	4x increase in loading	None	Fig. 2
2	increased loading	4x increase in loading	2x increase in aeration	Fig. 2
3	increased loading	8x increase in loading	None	Fig. 2
4	increased loading	8x increase in loading	2x increase in aeration	Fig. 2
5	no aeration	no aeration	None	Fig. 3
6	feed composition	standard loading with 17x HN_3_	None	Fig. 4

Manually increasing aeration was examined as a disruption mitigation strategy for increased loading. No aeration, 4x loading with 2x aeration, 8x loading, 8x loading with 2x aeration and 17x ammonia loading disruption experiments were conducted in the same system, in that order. The system was operated for a minimum of 5 d with standard loading between experiments. The 4x loading experiment was conducted in a second BAMBi system of identical configuration.

### 2.4 Chlorine demand measurement

Measurement procedures for chlorine demand were based on the Standard Methods 2350 for Oxidant Demand/Requirement protocol (APHA, 2017). Chlorine-demand-free glassware was utilized in all chlorine demand measurements. Glassware and polytetrafluoroethylene (PTFE)-lined lids were first cleaned with soap and water, then soaked in chlorine solution (100 mg CL_2_/L free chlorine in de-ionized water (DI) for 12 h and dried at 60 °C. Each measurement was prepared in a total volume of 31 mL in an ∼45 mL processing vial.

Sample was added to each processing vial such that the sample represented dilutions of 1 in 2; 1 in 10; 1 in 20; or 1 in 50 of the total volume of 31 mL. Phosphate buffer (final concentrations added 1.5 g/L Na_2_HPO_4_, 2.3 g/L KH_2_PO_4_), NaCl (final concentration added 0.1 g/L), free chlorine (final concentration added 2 mg Cl_2_/L) and DI water to reach final volume were added. The chlorine was added last to the processing vial by slowly pipetting the chlorine into the liquid phase after all other reagents were added and the vial was mixed. The chlorine stock was stored in the dark at 4 °C and returned immediately when not in use. Each processing vial was then gently mixed (swirled by hand) for about 10 s and then slowly inverted 5 times. The contents were gently poured into a 23 mL incubation vial and capped, leaving no air space. The incubation vials were then incubated in the dark for 12–18 h at 20 °C. The final free chlorine was measured using Hach kits (LCK 310, Hach, Loveland, Colorado, USA) employing the DPD (N,N-Diethylparaphenylenediamine) method.

Samples were processed in triplicate, and triplicate control vials were prepared for each measurement using the same procedure, without the addition of sample. Chlorine demand was calculated by subtracting the free chlorine concentration in the sample from the average free chlorine concentration in the control, and multiplying by the dilution of the sample.

The dilutions utilized for each sample were based on the estimated chlorine demand of previous baseline measurements (0.5–1 mg/L Cl_2_ demand) and the concentration of ammonia in the sample. The chlorine demand of ammonia was estimated (1 mg N/L = 7.6 mg Cl_2_/L demand) based on the stoichiometric oxidation of ammonia through monochloramine to N_2_ gas (Pressley et al., 1972). Dilutions were also based on maintaining a much higher chlorine concentration in the incubation vial compared to the ammonia concentration, so the relationship between chlorine demand and ammonia nitrogen was preserved (1 mg N/L = 7.6 mg Cl_2_/L demand). The minimum incubation ratio utilized in this study was 50 (mg Cl_2_)/(mg ammonia) with the exception of the 24 h data point in the experiment where the aeration was turned off (scenario 5). This sample was not processed correctly, at a ratio of 22 (mg Cl_2_)/(mg ammonia), which may have resulted in an under-measurement of chlorine demand. Chlorine demand was not accurately measured directly during the disruptive period of the experiment with increased ammonia loading (scenario 6) because it became too difficult to control for factors that can affect accurate chlorine measurements (pH, chloride concentrations, processing duration, signal strength at measurement). Chlorine demand during this period has been estimated from the ammonia concentration.

### 2.5 Dissolved organic carbon (DOC), assimilable organic carbon (AOC), anions and ammonia

All samples for DOC and AOC measurements were collected and processed in glass vials muffled at 450 °C for 12 h and capped with PTFE lined-lids. DOC was measured using an infrared detector calibrated to organic carbon standards (DOC-Labor Dr. Huber, Karlsruhe, Germany). AOC measurement procedure was based on an established protocol (Hammes and Egli, 2005). AOC samples (50 mL) were first filtered through prewashed (50 mL of DI water) 0.2 μm polyethersulfone filters (Pall Port Washington, New York, USA). Trace elements, Fe-solution, and buffer solution were added in accordance with Prest et al. (2016). 1 mL of a diverse bacteria inoculum was added, as previously described (Ziemba et al., 2018). The samples were mixed and then distributed equally into triplicate 45 mL vials, capped, incubated for 3–5 days at 30 °C on a dark 150 rpm shaker, and measured for total cell count (TCC). TCC was measured with a flow cytometer (Cytoflex, Bechman Coulter, Brea, California, USA) and SYBR® Green I stain (Life Technologies, Eugene OR, USA). Growth during incubation quantified as TCC was used to calculate AOC using the equation 1 μg AOC = 10^7^ cells (Hammes and Egli, 2005). Ion chromatography (Metrohm 881, Herisau, Switzerland) was used to measure the nitrite and nitrate in the samples, each with a minimum quantification limit of 0.2 mg N/L. Ammonia was measured using Hach kits (LCK 304, Hach, Love land, Colorado, USA) with a minimum quantification limit of 0.05 mg N/L.

## 3 Results

### 3.1 Increasing loading without changing composition

#### 3.1.1 Increasing loading produced a small increase in chlorine demand AOC and DOC, but not ammonia

**Fig. 2:**
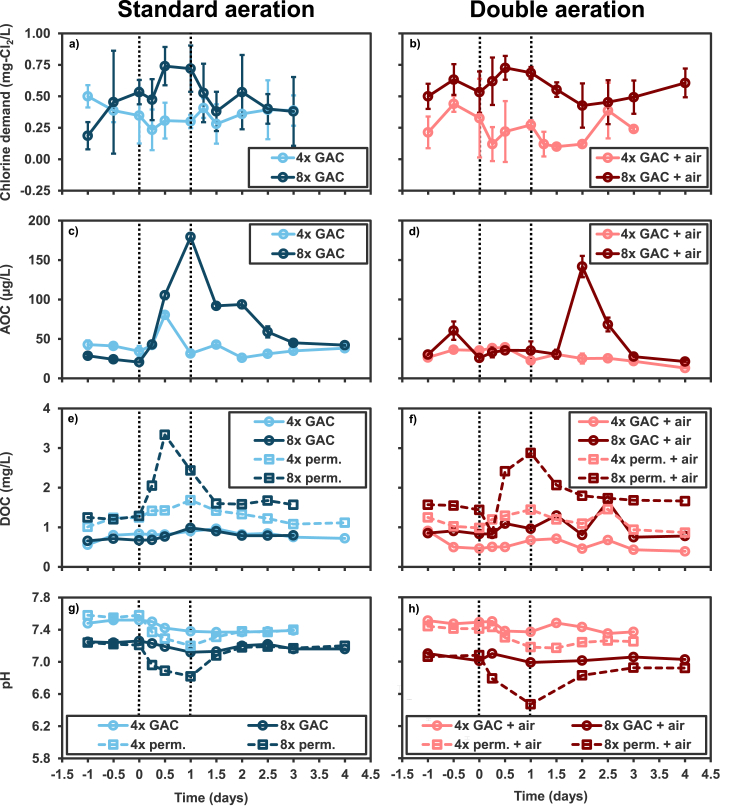
Chemical responses to 4x (scenario 1) and 8x (scenario 3) increase in loading (a,c,e,g) and 4x (scenario 2) and 8x (scenario 4) increases in loading with increased aeration (b,d,f,h). Perm. refers to permeate water that has just passed through the membrane while GAC refers to the water that has also passed through the GAC filter. The vertical dotted lines indicate the start and conclusion of increases in loading. Error bars indicate standard deviation of triplicate incubation vials for chlorine demand and AOC measurements.

The 4x increase in loading during a 1 d period (scenario 1) did not produce any clear increase in chlorine demand. The average chlorine demand observed during the 4x feeding experiment was 0.35 mg Cl_2_/L, which is comparable to the baseline values observed in the 8x feeding experiment (Fig. 2a). In contrast, the 8x increase in loading during a 1 d period (scenario 3) produced a peak chlorine demand of 0.74 mg Cl_2_/L ± 0.16 standard deviation. This peak was almost twice the average baseline chlorine demand measured before and following this disruption (starting at T_1 d_), of ∼0.4 mg Cl_2_/L.

DOC, AOC, pH and chemical concentrations were measured in water following treatment with the BAMBi (permeate) and after the GAC filter (GAC) to better understand the relative contributions of each step. The 4x feeding experiment (scenario 1) produced a DOC peak in the permeate at T_1 d_ (after 1 d, at the end of the disruption) of 1.7 mg/L, which is slightly higher than the baseline average of 1.15 mg/L ± 0.1 standard deviation. The 8x feeding experiment (scenario 3) produced a DOC peak at T_0.5 d_ (halfway into the disruption) of 3.3 mg/L, which is almost 3 times the baseline value before the increased feeding. Increases in DOC in the water following GAC were less pronounced.

Baseline values for AOC in the 4x and 8x feeding experiments were 36.5 ± 5.8 mg/L (average ± standard deviation) and 32.1 ± 11 mg/L respectively (Fig. 2e). A peak value of 80 ± 1.9 mg/L was observed in the 4x feed experiment (scenario 1) at T_0.5 d_ (halfway into the increased loading period), yet this is the only data point that appeared to be higher than the baseline. The 8x feeding experiment (scenario 3) produced a more pronounced increase to a peak of 179 ± 4.5 mg/L and a gradual return to baseline at T_3 d_. All ammonia measurements were below quantification (0.05 mg N/L) for the 4x and 8x loading experiments. Increasing loading generally reduced pH values, and these reductions were more significant in the permeate (∼0.4 pH units) relative to the effluent of the GAC (∼0.1 pH unit).

#### 3.1.2 Increasing aeration mitigated increases in AOC from increased loading, but did not mitigate increases in chlorine demand or DOC

Fig. 2 b, d, and f display changes in chlorine demand, AOC and DOC following increases of 4x or 8x in feed loading combined with 2x increases in aeration during the disruption period (scenarios 2 and 4 respectively). Increases in aeration did not produce different responses in chlorine demand or DOC. The maximum chlorine demand value achieved in scenarios 4 with 8x feed and 2x aeration (0.73 mg Cl_2_/L) was quite similar to scenario 3 with 8x feed with 1x aeration (0.74 mg Cl_2_/L). Increasing loading without increasing aeration either clearly increased AOC (8x feeding in scenario 3) or may have increased AOC (4x feeding in scenario 1). When the aeration was increased, neither condition resulted in any AOC increases during the disruption period. The 8x feeding with increased aeration (scenario 4) did produce elevated AOC measurements of 142 μg/L at T_2 d_ and 68 μg/L at T_2.5 d_, but this peak is not consistent with position on the experimental timeline, expected or observed in other scenarios.

### 3.2 Turning off aeration produces large increases in ammonia, AOC, DOC and chlorine demand

**Fig. 3:**
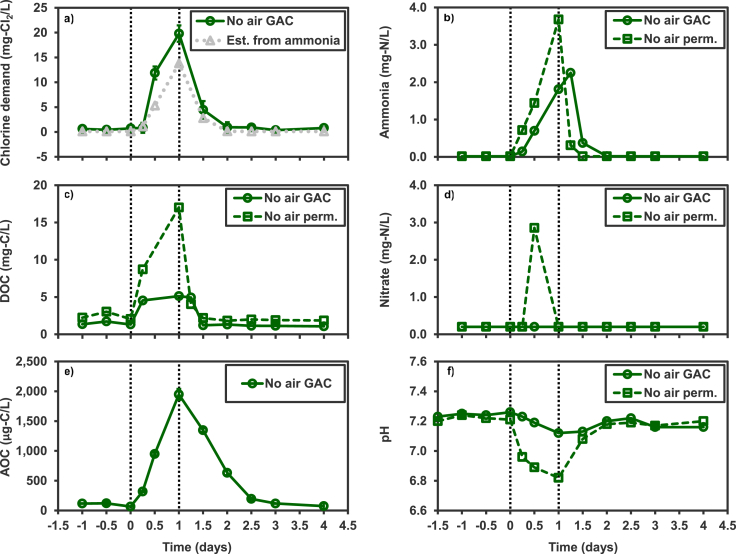
Chemical responses to turning off aeration (scenario 5). Values estimated (est.) from ammonia. were calculated based on the stoichiometric chlorine demand of ammonia. Perm. corresponds to permeate water that has just passed through the membrane while GAC corresponds to the permeate water that has also passed through the GAC filter. The vertical dotted lines indicate the start and conclusion of the disruption. The star beside the data point indicates that the presented value may be below the actual value due to measurement error. Ammonia and nitrate values measured below the minimum quantification limits are presented as the minimum quantification limits (0.05 mg N/L for ammonia, 0.2 mg N/L for nitrate). Error bars indicate standard deviation of triplicate incubation vials for chlorine demand and AOC measurements.

The increases in chlorine demand, AOC, DOC and ammonia were much greater following a 1 d period of shutting off aeration (scenario 5, Fig. 3) compared to an 8x increase in feeding (scenarios 3 and 4). The chlorine demand without aeration increased >30x from the average baseline value of 0.6 mg Cl_2_/L, to a peak likely greater than the measured value of 20 mg Cl_2_/L. This peak measurement was not conducted properly, and likely underestimated the true chlorine demand in the sample because the ratio of (ammonia)/(Cl_2_) in the incubation vials was not correct. Measured chlorine demand values are generally higher, across the experimental timeline, than what would be estimated stoichiometrically from the concentration of ammonia.

AOC concentrations increased almost 20x during the disruption to a peak value of 1947 μg/L ± 92 standard deviation, from a pre-disruption baseline of ∼100 μg/L. DOC concentrations increased in permeate and reached a peak of 17 mg/L up from a baseline concentration of 2.4 mg/L ± 0.52 standard deviation. The after GAC DOC concentration reached a peak of 5.1 mg/L up from a baseline concentration of ∼1.5 mg/L. The concentration of ammonia increased in both the permeate and GAC waters to reach peaks of 3.7 mg N/L at T_1 d_ and 2.3 mg N/L at T_1.25 d_ respectively. Nitrate (Fig. 3e) and nitrite (not plotted) were detected in the permeate at T_0.5 d_, but were below quantification (0.2 mg N/L) at all other time points in the permeate and at all time points in the GAC water. Baseline pH was approximately pH 7.2 in the permeate and GAC effluent, but dropped to respective minimums of pH 7.1 and 6.8 during the disruption (Fig. 3f).

### 3.3 A 17x increase in ammonia loading produced the largest increases in ammonia and chlorine demand, but no increases in AOC or DOC

**Fig. 4:**
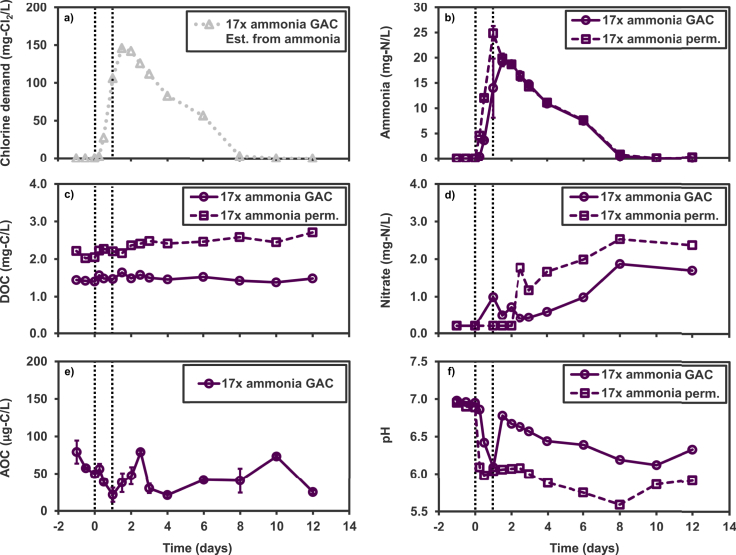
Chemical responses to increased ammonia loading in feed (scenario 6). Chlorine demand was estimated based on the stoichiometric chlorine demand of ammonia. Perm. corresponds to permeate water that has just passed through the membrane, while GAC corresponds to the permeate water that has also passed through the GAC filter. The vertical dotted lines indicate the start and conclusion of the increased-ammonia feeding. Ammonia and nitrate values measured below the minimum quantification limits are presented as the minimum quantification limits (0.05 mg N/L for ammonia, 0.2 mg N/L for nitrate). Error bars indicate standard deviation of triplicate incubation vials for AOC measurements.

Water quality responses to a 17x increase in ammonia loading (scenario 6) are presented in Fig. 4. Chlorine demand values for this disruption were estimated stoichiometrically from the concentration of ammonia, producing a peak chlorine demand of ∼146 mg Cl_2_/L, which is > 150 times greater than the pre-disruption baseline value of ∼0.9 mg Cl_2_/L. Ammonia concentrations in the GAC effluent peaked at T_1.5 d_, with a value of 19.2 mg N/L ± 0.89 standard deviation. The permeate reached a higher peak at T_1 d_, with a value of 24.8 mg N/L ± 1.4 standard deviation. Baseline values for ammonia were below quantification (0.05 mg N/L) in permeate and GAC effluent.

AOC concentrations did not display a clear trend in response to the ammonia feeding disruption. There was a decrease in AOC, initially in the baseline, that continued to a value of 21 μg/L at T_1 d_. Then the AOC increased to a maximum value of 79 μg/L. This value of 79 μg/L also matched the T_-1 d_ value. This range of AOC values is similar to the range of baseline AOC values observed testing other disruptive conditions. The DOC of the permeate increased over the course of the test from an initial value of 2.2 mg/L to a final value of 2.7 mg/L. Baseline values for pH were approximately 7 in the permeate and the GAC water. The pH of the permeate dropped to 6 at T_0.5 d_ and reached a minimum of 5.6 at T_8 d_. The pH of the GAC water dropped to 6.1 at T_1 d_, recovered to 6.8 at T_1.5 d_, but then declined again until T_10 d_.

## 4 Discussion

### 4.1 Increasing loading (without changing the composition) did not significantly disrupt system operation

The BAMBi + GAC system was effective at minimizing the impact of 4x and 8x loading increases when standard aeration was provided (scenarios 1 and 3). Our previous study, investigating TCC resulting from different concentrations of chlorine residual in recycled greywater, recommended chlorine dosing to achieve a chlorine residual of 1 mg Cl_2_/L, though an average value of 0.2 mg Cl_2_/L was sufficient to reduce TCC to drinking water levels (Ziemba et al., 2019). This higher setpoint (0.8 mg Cl_2_/L higher) was selected to provide a buffer to compensate for expected variation in chlorine demand under standard conditions. In our 8x loading disruptive testing (scenario 3), the buffer of 0.8 mg Cl_2_/L would have been more than sufficient to overcome the additional chlorine demand added (+0.25 mg Cl_2_/L).

The dominant removal mechanism in the BAMBi is biological metabolism (Künzle et al., 2015; Ravndal et al., 2015). GAC is capable of removing carbon-containing contaminants through a combination of biological conversion and physical sorption. A mass balance performed on the 8x loading disruption (scenario 3), demonstrated that the GAC provided only polishing, removing approximately 2% of the additional carbon loading. The remaining 98% of the carbon removal can be attributed to the BAMBi. As 4x and 8x increases in loading did not change the composition of the feed, the existing bacterial community was likely well-suited to remove the additional material.

The limited impacts on DOC and AOC during 4x and 8x increases in loading indicates that oxygen limitation may not have been significant. In contrast, significant oxygen limitation during the no aeration disruption test (scenario 5) was likely responsible for more significant DOC accumulation (above 15 mg DOC/L in the permeate). Increasing aeration during the 4x and 8x increases in loading (scenarios 2 and 4) did not produce improvements in performance in DOC removal compared to standard aeration (scenarios 1 and 3). This further supports the case that the standard aeration of 6 L/min may be significantly more than what is needed under standard loading.

### 4.2 Discontinued aeration and/or increased ammonia loading can disrupt system operation

In a system that relies on maintaining a free chlorine residual, the chlorine demand concentrations produced by our examined aeration (scenario 5) and ammonia loading (scenario 6) disruptions (peaking at ∼30 and ∼146 mg Cl_2_/L respectively) would overcome reasonable chlorine dosing strategies.

The accumulation of ammonia and DOC during the no aeration testing (scenario 5) indicates a collapse of biological treatment, likely driven by oxygen limitation. When the ratio of Cl_2_:(NH_3_) is high, ammonia produces chlorine demand at a ratio of 7.6:1 (mg Cl_2_/L:mg [NH_3_ N/L]) (Pressley et al., 1972). Using this relationship, we can estimate that approximately half the chlorine demand produced during the no aeration test can be attributed to ammonia. The increase in DOC contributes to the chlorine demand not explained by ammonia. Even without aeration, approximately half of the ammonia added to the system was removed by the end of the no aeration disruption. The removal of ammonium was likely a combined effect of volatilization, nitrification driven by oxygen that could enter the system and water removed from the system to maintain volume. The recirculation of water through the system (∼120 L/d) would have provided a limited opportunity for volatilization and oxygen ingress. The absence of nitrite and nitrate at the end of the disruption indicated that denitrification was also active.

Increased ammonia loading (scenario 6) led to an acute accumulation of ammonia in the system. Some ammonia was likely lost due to volatilization, some was utilized in heterotrophic growth, some was removed from the system while maintaining volume and the balance was likely oxidized through nitrification (decreasing the pH). The reduction in pH, resulting from limited alkalinity, may have negatively impacted further nitrification, though heterotrophic metabolism did not appear hindered, with DOC and AOC in the permeate virtually unchanged during the disruption. The eventual recovery of the pH and the ultimate removal of nitrogen from the system was likely driven by denitrification. Biological activity within the GAC likely contributed to this denitrification, as passage of water through the GAC resulted in lower nitrate concentrations and increased pH.

Testing failure of aeration and ammonia overloading demonstrated that the biological processes in the BAMBi can be disturbed to a level where downstream chlorine-based disinfection cannot keep up. Unlike chlorine that reacts with organics, the chlorine in scenarios 5 and 6 that reacts with ammonia can still contribute to anti-microbial value through the formation of chloramines. Inactivation of disperse bacteria with chloramines requires extremely high concentration time (CT) values compared to free chlorine (Gray 2014). For example, two-log inactivation of *Escherichia coli* requires 0.119 (mg/L)•min CT value with free chlorine, but 11 (mg/L)•min CT value with monochloramine (Baker et al., 2002). Any changes in pH that occur while free chlorine is present, will also significantly impact the inactivation potential of a given free chlorine concentration. Free chlorine in water is an equilibrium between hypochlorous acid (HOCl) and hypochlorite (ClO^−^), with a pKa of approximately 7.5. Shifting the pH lower increases the concentration of HOCl, which is approximately 80 times more effective at disinfection (Gray 2014).

### 4.3 Designing a system to overcome disruptions

The current BAMBi + GAC system, when operated as intended, encounters very little risk of failure from practical increases in nutrient-balanced loading. This is, in part, due to the amount of water that can be recycled in the BAMBi being limited. The flow of water through the membrane was observed to be approximately 120 L/d under standard operating conditions. Demanding hand washing water at a higher rate than 120 L/d would lead to exhaustion of the clean water tank, and limit the rate of further inputs. If the material contributed per hand washing event is consistent, then the maximum daily water flux would limit the loading to 2x. While ensuring that no foreign substances (anti-bacterial agents, acids or bases, membrane-fouling materials) are added to the BAMBi is an essential responsibility of the user, washing hands in a manner that contributes significantly more loading than expected should also be prevented. It is also essential to limit excessive ammonia loading on the BAMBi, including urine.

Previous research has demonstrated improved removal of target compounds in biological greywater treatment systems by supplementing nutrients in which the system was believed to be deficient (Jefferson et al., 2001; Ziemba et al., 2018). The current study utilizes a combination of ammonia and nitrate stoichiometrically added for growth on the organic carbon in our synthetic feed. Bacteria generally prefer ammonia as a source of nitrogen, and grow faster on ammonia than any other nitrogen source (Merrick and Edwards, 1995), but we have demonstrated that excess ammonia loading can pass through BAMBi and through the GAC to introduce significant chlorine demand to the final water product. Excessive ammonia on a regular basis may also promote a population of nitrifying bacteria, which may enable the system to better overcome excessive ammonia loading in the future. We suggest further research to better understand how effectively nitrate can serve as a nitrogen source (without contributing chlorine demand) and also how conditioning a population (for example by supplementing ammonia to support nitrifier growth) can better enable a biological system to overcome disruptions.

In a recent field study, we tested a handwashing water recycling system in Durban, South Africa, with a similar setup as presented in this study but with a design flow of 500 L/day and including an electrolysis post-treatment for the production of chlorine (Reynaert et al., 2020). In spite of highly variable nitrogen concentrations in the greywater input (between 14 and 260 mg total N/L), the treated water always contained residual chlorine under normal operation and ammonia concentrations were mostly below the detection limit of 0.05 mg N/L. The only disruption during the field test was due to a prolonged power outage (no aeration and no chlorine production), which resulted in the consumption of all free chlorine and 1.3 mg N/L ammonia in the treated water. Although the effect of variable ammonia concentrations on the treatment performance was not the focus of this field tests, the results support that nitrifying bacteria are capable of adapting to variable ammonia inputs.

Overall system robustness can be improved by implementing redundant systems, such as aeration pumps and/or air distribution hardware (diffusion tubing or stones), to help overcome component failure. Battery backup or manually-operated aeration systems could overcome power outages. Adding additional treatment steps can also mitigate the impacts of disruptions. Alternative disinfection technologies, such as UV, have demonstrated anti-bacterial performance without chlorine and independent of chlorine demand (Beck et al., 2013; Gilboa and Friedler, 2008). Electrochemical oxidation is one potential strategy to oxidize ammonia (Li and Liu, 2009) and organic material (Martínez-Huitle et al., 2015) that contribute to chlorine demand. We have previously demonstrated how adding electrochemical oxidation to the BAMBi either may (Ziemba et al., 2020), or may not significantly impact organic carbon, in favor of chlorine production (Ziemba et al., 2019). Differences in results were driven by variations in hardware configurations for the electrochemical units, but also by the composition of the wastewaters being treated. In this study, we have elected to investigate the impacts of these disruptions using only the basic BAMBi + GAC system without electrolysis, which has allowed us to identify for which circumstances additional treatment could be productive.

Finally, user safety should be ensured by smart system operation where problems in the system are detected and the system automatically prevents user access until operation is back to normal. Smart operation can include sensing of system components (e.g., pumps) and simple sensors (e.g., redox potential in the clean water tank) as indicators for normal operating conditions.

We have focused our disruption analysis on chlorine demand, but if we look more broadly at system stability, we see that the BAMBi + GAC system was able to recover from each of these disruptions once the standard operating conditions were resumed. The biological processes were impacted with each disruption, but the microbial community was not killed, the membrane did not foul and the water flux did not stop. We have suggested practical strategies that can improve the microbial safety for the user even under challenging conditions, and we believe further development is justified because of the simplicity, affordability and robustness of the core processes of the BAMBi + GAC system. This future development would include an evaluation of the effectiveness of the strategies we have identified in this paper and ultimately a quantitative microbiological risk assessment (QMRA) to demonstrate comprehensive microbial safety.

## 5 Conclusions

● The BAMBi + GAC treatment system delivers robust performance against increases in loading, when the ratio of ammonia to carbon in the loading is maintained.
● Interruptions in aeration or increases in ammonia loading may produce chlorine demands sufficient to overcome any practical chlorine dosing strategy due to passage of ammonia and/or organic materials through the system.
● Ammonia loading is controlled by user behavior, geographic and social considerations, but also through potential nutrient-balancing strategies. Higher ammonia loading than required for heterotrophic growth may risk increases in chlorine demand when the ammonia is not biologically consumed.
● Maintaining microbial safety in greywater recycling is a complex and application-specific task, requiring hardware designed to overcome challenging operation, but also effective communication to minimize inappropriate operation, and ensure understanding of limitations.

## Appendix: Research data

Research data for this article are available at https://doi.org/10.25678/0002ZN.

## Declaration of competing interest

The authors declare that they have no known competing financial interests or personal relationships that could have appeared to influence the work reported in this paper.
